# ATM activation accompanies histone H2AX phosphorylation in A549 cells upon exposure to tobacco smoke

**DOI:** 10.1186/1471-2121-8-26

**Published:** 2007-06-26

**Authors:** Toshiki Tanaka, Xuan Huang, Ellen Jorgensen, Diana Gietl, Frank Traganos, Zbigniew Darzynkiewicz, Anthony P Albino

**Affiliations:** 1 Brander Cancer Research Institute and Department of Pathology, New York Medical College, Valhalla, NY, 10595, USA; 2 Vector Research Ltd., 712 Fifth Ave., New York, NY 10019, USA

## Abstract

**Background:**

In response to DNA damage or structural alterations of chromatin, histone H2AX may be phosphorylated on *Ser*139 by phosphoinositide 3-kinase related protein kinases (PIKKs) such as *ataxia telangiectasia *mutated (ATM), ATM-and Rad-3 related (ATR) kinase, or by DNA dependent protein kinase (DNA-PKcs). When DNA damage primarily involves formation of DNA double-strand breaks (DSBs), H2AX is preferentially phosphorylated by ATM rather than by the other PIKKs. We have recently reported that brief exposure of human pulmonary adenocarcinoma A549 cells or normal human bronchial epithelial cells (NHBE) to cigarette smoke (CS) induced phosphorylation of H2AX.

**Results:**

We report here that H2AX phosphorylation in A549 cells induced by CS was accompanied by activation of ATM, as revealed by ATM phosphorylation on *Ser*1981 (ATM-S1981^P^) detected immunocytochemically and by Western blotting. No cell cycle-phase specific differences in kinetics of ATM activation and H2AX phosphorylation were observed. When cells were exposed to CS from cigarettes with different tobacco and filter combinations, the expression levels of ATM-S1981^P ^correlated well with the increase in expression of phosphorylated H2AX (γH2AX) (R = 0.89). In addition, we note that while CS-induced γH2AX expression was localized within discrete foci, the activated ATM was distributed throughout the nucleoplasm.

**Conclusion:**

These data implicate ATM as the PIKK that phosphorylates H2AX in response to DNA damage caused by CS. Based on current understanding of ATM activation, expression and localization, these data would suggest that, in addition to inducing potentially carcinogenic DSB lesions, CS may also trigger other types of DNA lesions and cause chromatin alterations. As checkpoint kinase (Chk) 1, Chk2 and the p53 tumor suppressor gene are known to be phosphorylated by ATM, the present data indicate that exposure to CS may lead to their phosphorylation, with the downstream consequences related to the halt in cell cycle progression and increased propensity to undergo apoptosis. Defining the nature and temporal sequence of molecular events that are disrupted by CS through activation and eventual dysregulation of normal defense mechanisms such as ATM and its downstream effectors may allow a more precise understanding of how CS promotes cancer development.

## Background

The ATM protein kinase is a key component of the signal transduction pathway that is activated in response to DNA damage, particularly if the damage involves formation of DNA DSBs [1-7]. ATM activation takes place through an autophosphorylation site on *Ser*1981, which leads to dissociation of the inactive ATM dimer (or higher-order multimer) into single protein molecules with kinase activity [3,4]. While *Ser*1981 phosphorylation is responsible for dimer dissociation, the catalytic domain of ATM, which becomes accessible to substrates only when ATM is a monomer, is located outside of the *Ser*1981 locus [3]. ATM activation is mediated through mobilization of the Mre11-Rad50-Nbs1 (MRN) complex, which recognizes DNA damage, recruits ATM and also functions in targeting ATM to initiate phosphorylation of the respective substrates [2,6-9]. The initial activation of ATM occurs at some distance from the site of DSB and is initiated by the changes in higher order chromatin structure resulting from relaxation of the topological stress of the DNA double helix following induction of the DSB [4]. It should be noted, however, that the mechanism of ATM activation is more complex than just via autophosphorylation. Recent evidence suggests activation requires prior ATM acetylation, which is mediated by the Tip60 histone acetyltransferase [10], and is associated with protein phosphatase 5 activity [11], as well as interactions with other factors [12].

Several downstream target substrates phosphorylated by ATM, such as p53 (TP53), Chk2, Chk1, Brca1 and H2AX, are recognized as having tumor suppressor functions [4], since their phosphorylation is essential for successful DNA repair, suppression of cell progression through the cell cycle and/or induction of apoptosis. Disruption in one or more of these mechanisms can promote expansion of DNA damage to cell progeny and instigate genomic instability. Development of the phospho-specific antibody towards *Ser*1981 phosphorylated ATM (ATM-S1981^P^) provided a convenient tool to detect ATM activation immunocytochemically in tissue sections [13] and to measure expression of ATM-S1981^P ^in individual cells by cytometry [14,15]. It is noted that when activation of ATM is induced by DSBs, the activated ATM is localized in individual discrete foci [16]. However when other DNA lesions or chromatin alterations trigger ATM activation, the activated ATM is diffusely scattered throughout the nucleoplasm and does not form distinct foci [3,17].

One substrate phosphorylated by ATM is a variant of histone H2A, histone H2AX [2]. Its phosphorylation on *Ser*139 occurs in response to induction of DSBs. Expression of phosphorylated H2AX [termed γH2AX [18-20]] is, therefore, considered to be a marker of induction of DSBs [18-22]. As with ATM-S1981^P^, the development of a phospho-specific γH2AX antibody made it possible to study H2AX phosphorylation in individual cells [18-20], and to quantify by cytometry γH2AX expression in response to DNA damage by different agents [21-25].

H2AX can be phosphorylated not only by ATM but also by other phosphoinositide 3-kinase related protein kinases (PIKKs) such as ATM- and Rad3-related (ATR), or DNA dependent protein kinase (DNA-PKcs) [5,26,27]. For example, H2AX phosphorylation in response to replication stress induced by hydroxyurea, excess thymidine or aphidicolin was shown to be mediated by ATR [28] rather than by ATM [29]. In contrast, DNA-PKcs was shown to mediate H2AX phosphorylation in hypertonically-stressed cells [30], as well as in response to DNA fragmentation during apoptosis [31]. DNA fragmentation in apoptotic cells was also seen to be accompanied by activation of ATM, which would suggest that ATM may mediate H2AX phosphorylation during apoptosis as well [29,32]. In addition, there is also evidence that in response to DNA damage caused by ionizing radiation, all three PIKKs (i.e., ATM, ATR and DNA-PKcs) may function redundantly to phosphorylate H2AX [33,34]. Presumably, each PIKK responds to a hierarchy of specific types of DNA damage or cell contexts in which it may alternately play a primary or supporting role, or no role at all, in the phosphorylation of H2AX. To date, most published data implicate ATM as the dominant physiologically mediator of H2AX phosphorylation in response to DSB formation [2,4,6-9].

We have recently reported that brief exposure of human normal bronchial epithelial cells and human pulmonary adenocarcinoma A549 cells to CS or to cigarette smoke condensates induced H2AX phosphorylation [24]. In a subsequent study we observed that the reactive oxygen species (ROS) scavenger N-acetyl-L-cysteine (NAC) was, to a large extent, able to protect the cells from induction of H2AX phosphorylation by CS [35]. The data suggested that CS-induced DNA damage in these cells involves formation of DSBs, and that these lesions were mediated, in part, by ROS. The present study was aimed to reveal whether ATM is activated by exposure of cells to CS. If ATM activation preceded or was concurrent with H2AX phosphorylation, and if it correlated in intensity with H2AX phosphorylation, it would strongly suggest that ATM is the PIKK that phosphorylates H2AX in response to cell exposure to CS. It would also provide clues as to which potential downstream cell cycle checkpoints, whose activation is mediated by ATM [3,4], may be impacted by CS.

## Results

**Figure 1:**
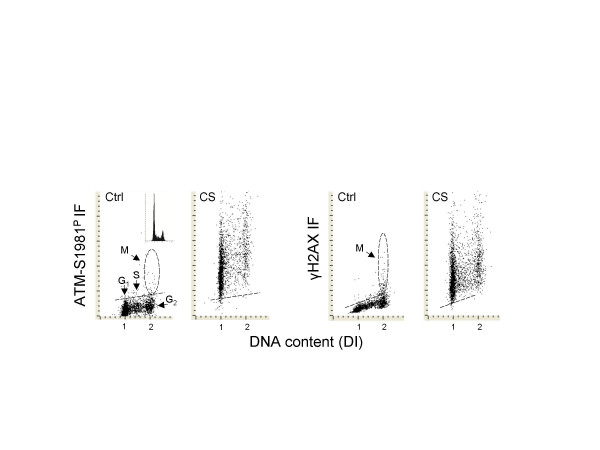
**Induction of ATM activation and H2AX phosphorylation in A549 cells following their exposure to IM16 CS**. Bivariate distributions (scattergrams; DNA content, DI *vs. *ATM-S1981^P ^IF, or *vs*. γH2AX IF, respectively) of the A549 cells, mock treated (Ctrl) or exposed 20 min to CS (CS), as described in the Materials and Methods. After exposure to CS or mock treatment the cells were incubated for an additional hour, then fixed and subjected to immunocytochemical detection of ATM-S1981^P ^or γH2AX, their DNA counterstained with DAPI and the intensity of IF- and DAPI-fluorescence measured by iCys. The inset shows the cellular DNA content histogram representing cells from these cultures. Based on differences in DI the cells can be subdivided into G_1_, S and G_2_M compartments, as shown in the left panel. Mitotic (M) A549 cells constitutively express higher level of ATM-S1981^P ^and γH2AX [14,38,70,71] and their position on the scattergrams is marked within dashed oval boundaries. The dashed line represents the upper threshold of ATM-S1981^P^or γH2AX IF level for 97% of interphase cells.

Exposure of A549 cells to CS led to a marked rise in expression of ATM-S1981^P ^and γH2AX (Fig. 1). The bivariate analysis of cellular DNA content and intensity of immunofluorescence made it possible to correlate expression of ATM-S1981^P ^or γH2AX with cell position in the cell cycle. Expression of ATM-S1981^P ^and γH2AX was markedly increased in cells exposed to CS compared to the mock treated cells. In fact, nearly all cells from the CS-treated cultures expressed ATM-S1981^P ^or γH2AX IF above the level representing the upper threshold of expression of these phospho-antibodies for 97% of the cells from the mock-treated cultures. It is also apparent from the raw data shown in the scattergrams (Fig. 1), that exposure to CS led to induction of ATM-S1981^P ^or γH2AX expression at a comparable level in all phases of the cell cycle.

**Figure 2:**
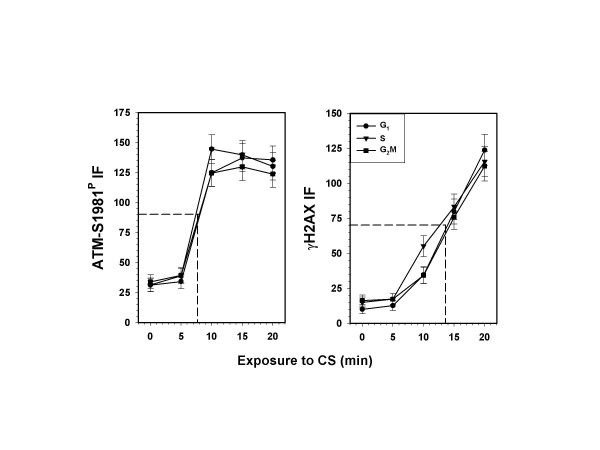
**IM16 CS-dose (time of treatment) dependence in induction of ATM-S1981^P ^and γH2AX for A549 cells at different phases of the cell cycle**. The mean values of ATM-S1981^P ^and γH2AX IF for populations of G_1_, S, and G_2_M cells (± SEM) from the mock-treated sample (0 min) and from the samples treated for 5, 10, 15 and 20 min with CS, were estimated by gating analysis on the bivariate distributions as shown in Fig. 1. The mid-slope points are projected on the CS dose (time of exposure) coordinate. It should be noted that similar curves of γH2AX IF, determined under identical conditions, were previously published [35]. The present curves for γH2AX IF, though virtually identical to the previously published data, represent new sets of slides in which γH2AX IF was measured in one chamber while ATM-S1981^P^was measured in the second chamber of the same slide.

Fig. 2 presents the quantitative data illustrating the CS-dose dependence of the induction of ATM-S1981^P ^and γH2AX in A549 cells in relation to cell position in the cell cycle. As is evident, the time-dependent increases in ATM-S1981^P ^and γH2AX IF were essentially the same for all cells, regardless of the cell cycle phase. While the maximal (plateau) rise in ATM-S1981^P ^expression was achieved after 10 min exposure to CS, induction of γH2AX continued to increase during the 20 min exposure. The mid-slope increase in induction of ATM-S1981^P ^and γH2AX was at 8 and 13 min of exposure to CS, respectively. Caspase-3 activation was monitored on parallel slides to rule out any possible effects of apoptosis on induction of either γH2AX or ATM-S1981^P ^IF. No apoptosis was observed under the experimental conditions used in this study (data not shown).

**Figure 3:**
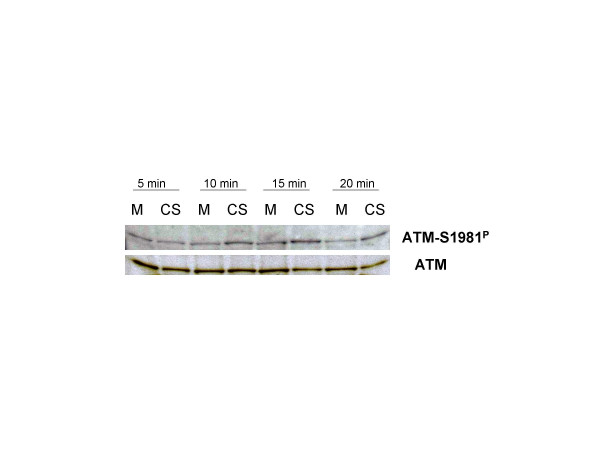
**Induction of ATM activation in A549 cells by CS as revealed by Western blotting**. The cells were exposed to 5, 10, 15 or 20 min of IM16 smoke (CS) and harvested at 30 min post-exposure. The extent of ATM activation (ATM-S1981^P^) was determined by Western blotting and compared with the respective samples of the mock treated cells (M) and with expression of ATM detected by antibody that was not phospho-specific.

The immunocytochemical/cytometric data demonstrating induction of ATM-S1981^P ^as a function of dose of CS (time of exposure) were confirmed by Western blotting (Fig. 3). The data show that 10, 15 and 20 min exposure to CS led to an increase in intensity of the ATM-S1981^P ^bands compared to the respective bands from the mock-treated cells. The bands detected by the ATM antibody that were not phospho-specific do not show changes in intensity between CS treated and untreated cells.

**Figure 4:**
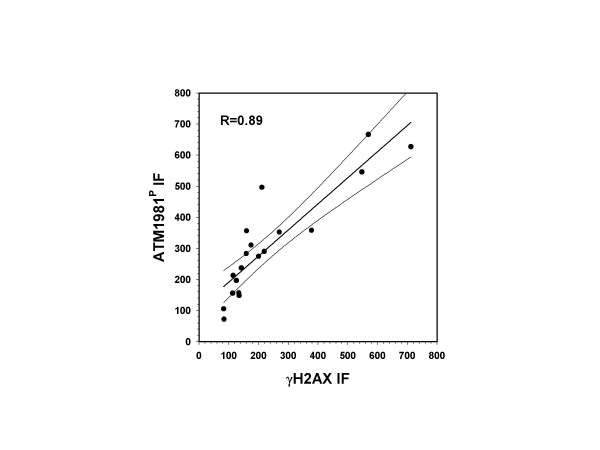
**Correlation between induction of expression of ATM-A1981^P ^and γH2AX in A549 cells following exposure to CS**. Cells were exposed to CS from cigarettes with different tobacco and filter combinations. Each sample was processed in parallel to measure expression of ATM-S1981^P ^and γH2AX and the data are presented as the regression plot with 95% confidence limits. The correlation coefficient R = 0.89.

In order to determine if there was a direct quantitative relationship between γH2AX IF induction and ATM-S1981^P ^activation, cells were exposed to CS from cigarettes with different tobacco and filter combinations (Albino et al., manuscript in preparation). Fig. 4 shows in fact that there is a good correlation (R = 0.89) between these two variables suggesting that the expression levels of γH2AX and ATM are directly linked.

**Figure 5:**
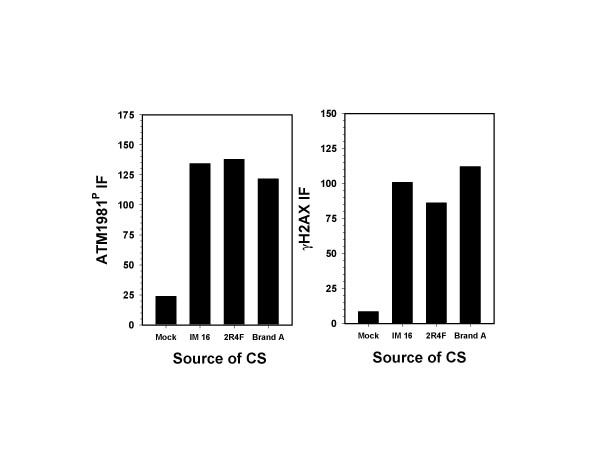
**Induction of ATM phosphorylation and H2AX phosphorylation by CS from different cigarettes**. A549 cells were exposed under identical conditions to CS from IM16 reference research cigarette (FTC values of 15.7 mg "tar" and 1.01 mg nicotine), 2R4F reference research cigarette (FTC values of 9.7 mg "tar" and 0.85 mg nicotine), or Brand A (FTC values of 15 mg "tar" and 1.1 mg nicotine). The IM16, 2R4F reference cigarettes, and the Brand A commercial cigarette had essentially equivalent levels of ATM-A1981^P ^and γH2AX IF.

It was of interest to compare standard reference cigarettes to a commercial brand in terms of the level of expression of ATM-S1981^P ^and γH2AX. We therefore examined two industry standard reference cigarettes, IM16 (FTC values of 15.7 mg "tar" and 1.01 mg nicotine) and 2R4F (FTC values of 9.7 mg "tar" and 0.85 mg nicotine), and a leading commercially available U.S. cigarette brand, designated here as Brand A (FTC values of 15 mg "tar" and 1.1 mg nicotine). When cells were exposed for 20 min to CS from either IM16, 2R4F, or Brand A commercial cigarettes, there was little difference in the degree of γH2AX IF or ATM-S1981^P ^expression (Fig. 5).

Caffeine and Nu7026 are putative inhibitors of ATM [36] and DNA-PKcs [37], respectively. Either of these kinases can potentially cause the phosphorylation of H2AX following DNA damage. To explore their potential inhibitory effect 4 mM caffeine or 10 μM Nu7026 was added to the medium 1 h prior to exposure to CS, was present in D-PBS during exposure to CS from IM16 cigarettes and, further in the medium during the 1 h post-exposure incubation.

**Figure 6:**
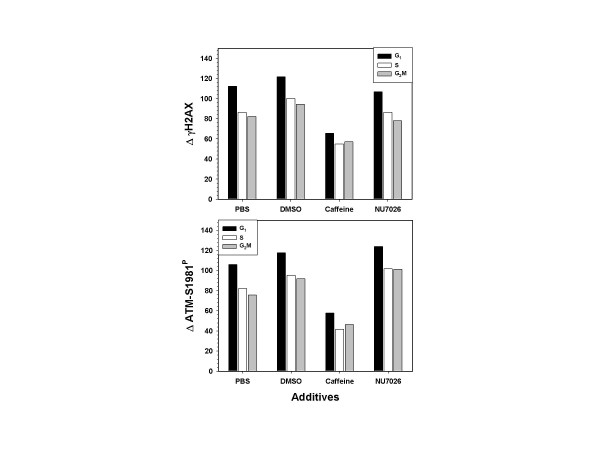
**Effect of treatment with caffeine and NU7026 on ATM and H2AX phosphorylation**. A549 cells were exposed to CS for 20 min either in the presence of PBS, 4 mM caffeine or 10 uM NU7026 or were collected in the absence of smoke treatment (mock exposure). Cells exposed to caffeine and NU7026 were also treated for one hour prior to exposure and one hour subsequent to cell harvest. Only treatment with caffeine lowered the level of expression of ATM-A1981^P ^and γH2AX IF.

As can be seen in Figure 6, CS from IM16 induced a considerable level of γH2AX IF in A549 cells. The values in the figure represent the difference between the CS-treated sample and the mock-treated control (Δ). Inclusion of the vehicle DMSO had no appreciable effect on the level of γH2AX IF generated by CS from IM16. Caffeine, at a concentration of 4 mM, reduced the average level of γH2AX IF by approximately 37%. In parallel cultures on the same slide, ATM-S1981^P ^IF was reduced by 45% in the presence of caffeine. Nu7026 had little or no inhibitory effect on γH2AX IF (reduced by 3%) while ATM-S1981^P ^IF was actually increased slightly (Fig. 6). No cell cycle phase specificity associated with the caffeine-induced decrease in either ATM-S1981^P ^or γH2AX IF was apparent.

**Figure 7:**
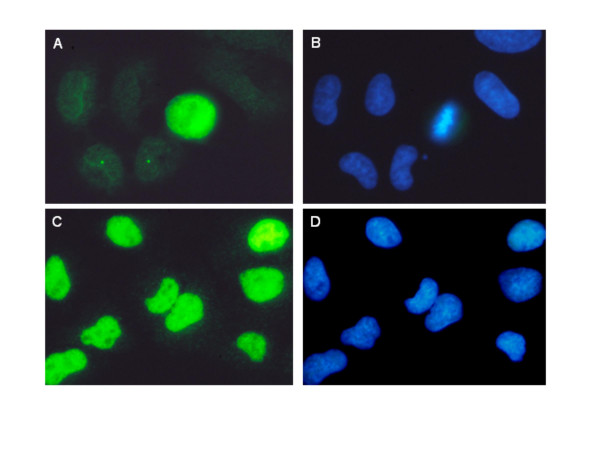
**Induction of ATM activation in A549 cells following exposure to CS**. Photomicrographs of the mock treated cells (A, B) or cells exposed for 20 min to IM16 CS (C, D). The cells were immunocytochemically labeled with phospho-specific ATM-S1981^P ^antibody, with the secondary antibody fluorescing green (A, C); their DNA was counterstained with DAPI (B, D). In the mock treated sample (A) only the mitotic cell is strongly ATM-S1981P positive. The punctuate staining in two other cells represents labeling of their centrosomes, consistent with data previously reported [14,38,70,71]. The cells exposed to CS exhibited strong and rather uniform IF over the entire nucleus. Nikon Microphot FXA, Obj. 60×

**Figure 8:**
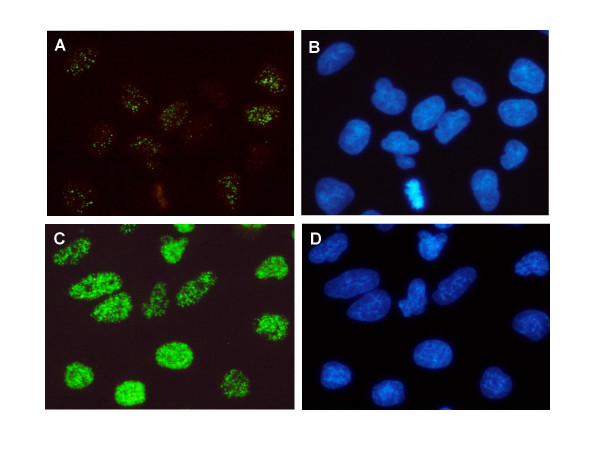
**Induction of H2AX phosphorylation following exposure to CS**. Photomicrographs of the mock treated cells (A, B) or cells exposed for 20 min to IM16 CS (C, D). The cells were immunocytochemically labeled with phospho-specific γH2AX antibody, with the secondary antibody fluorescing green (A, C); their DNA was counterstained with DAPI (B, D). Note the low level of constitutive H2AX phosphorylation in the mock-treated cells with discrete punctuate pattern reflecting the presence of IF foci, consisted with the data presented before [70,77]. The CS treated cells show intense γH2AX IF due to very large numbers of IF foci located close to each other. Photomicrographs of the CS treated cells were obtained using automatic camera exposure. To demonstrate the much lower γH2AX IF of mock treated cells, the camera aperture was opened and closed manually for an equivalent length of time.

Figs. 7 and 8 reveal the effect of CS on expression of ATM-S1981^P ^and γH2AX detected immunocytochemically in individual A549 cells and observed by fluorescence microscopy. In the mock-treated sample only the mitotic cell shows strong ATM-S1981^P ^IF which is localized throughout the whole cell (Fig. 7A). Some interphase cells show the presence of ATM-S1981^P ^in single point-like structures, which were previously identified as centrosomes [38]. Utilizing expression of ATM-S1981^P ^in the mitotic cell to determine the duration of exposure for the photograph, the background, constitutive expression of ATM-S1981^P ^in the interphase cells of the untreated sample is barely visible. In the cells exposed to CS, there is strong and rather uniform IF revealing the presence of activated ATM throughout the nucleus (Fig. 7C).

The presence of γH2AX in the mock treated cells appears in the form of distinct foci that are randomly distributed through the nuclei (Fig. 8A). The number of such foci and their proximity are greatly increased in the cells exposed to CS, which leads to much stronger overall intensity of γH2AX IF (Fig. 8C).

## Discussion

CS is a complex chemical mixture of gases and suspended particulate matter that contains a wide range of carcinogens, mutagens, and free radicals that can induce an array of DNA lesions (e.g., adducts, deletions, insertions, point mutations, structural chromosomal aberrations, aberrant sister chromatid exchanges, micronuclei, and single strand DNA breaks) that have been strongly implicated in the development and progression of human cancers by epidemiological studies [39-48]. Our previous studies showed that CS and cigarette smoke condensate (i.e., 'tar') can also directly induce DSBs in both normal and malignant lung cells [24,35]. DSBs are a major type of DNA damage that can lead to translocations and chromosomal instability, two important mechanisms in the generation of malignant tumors [49]. Consequently, detection of DSBs and the various proteins that sense and orchestrate repair of these lesions may ultimately provide a more precise measurement of the potential cancer risk in individuals exposed to CS.

In the current study, we show that exposure of A549 cells to CS led to concurrent activation of ATM and phosphorylation of H2AX. Moreover, there was a close relationship between ATM activation and H2AX phosphorylation both in timing (dose) and in the degree of response to CS. Thus, both events were triggered after 10 min exposure to CS (Fig. 3), and the degree of response, when cells were treated with CS from cigarettes with different tobacco and filter combinations, was strongly correlated (Figs. 4 and 5). In addition, both ATM activation and H2AX phosphorylation were partially suppressed by caffeine but not by NU7026, implying that CS-induced H2AX phosphorylation was predominantly mediated by ATM. DNA damage that primarily results in formation of DSBs is believed to trigger H2AX phosphorylation mediated by ATM rather than by ATR or DNA-PKcs [2,4,6-9,50]. This observation is in contrast to other genotoxic agents such as hydroxyurea, aphidicolin, and UVR that induce H2AX phosphorylation as a function of replication stress, but which is mediated by ATR and not by ATM [28,29]. However, despite the apparent causal relationship between ATM activation and H2AX phosphorylation in CS-treated A549 cells, a definitive study in which ATM expression is suppressed by interfering RNAs (RNAi) is currently being conducted in order to prove conclusively that ATM is the primary responsive kinase.

How might CS induce DSBs? The most common mechanism by which DSBs are thought to be generated is as a result of stalled DNA replication forks at the sites of single strand DNA lesions such as single-strand breaks (SSBs) during DNA replication [51]. Unrepaired DSBs generated by such mechanisms may result in chromosome breakage and mironucleation leading to genetic instability and malignant transformation [52,53]. However, although it is clear that CS can cause numerous SSBs that may induce replication fork stalling [47,54], some data suggest that even large amounts of SSBs are incapable of provoking ATM activation [50]. Whether this is a general phenomenon or restricted to a specific cell type or cell context remains to be determined, but it may suggest that the induction of DSBs by CS occurs via a mechanism other than SSB formation. An important observation in this regard is that if stalled replication forks at SSB lesions were the dominant mechanism of DSB formation, it would be expected to reflect a characteristic pattern of H2AX phosphorylation that is distinctly maximal in S-phase cells [27,55]. While we observed somewhat higher levels of H2AX phosphorylation in S-phase cells compared to G_1 _cells upon exposure to CS [see Fig 2 and [24,35]], the difference was rather small. These data imply that the contribution of this mechanism to the overall level of DSB damage, as reflected by the increased expression of γH2AX, was not predominant. One conjecture to mechanistically explain this observation is the possible activation of SMC1, a critical downstream event in the ATM-NBS1-BRCA1 pathway that engages the S-phase checkpoint and prevents movement of the replication forks and thus their collisions with ssDNA lesions [17,56,57]. However, whether SMC1 is activated in CS-exposed cells remains to be determined.

CS also contains a range of carcinogens known to result in covalently linked DNA adducts either directly or indirectly after metabolic activation [58]. Upon cell proliferation, these type of adducts can induce DNA replication errors that may result in carcinogenic events [59,60]. Moreover, as these DNA-carcinogen adducts are removed by base excision repair mechanisms, defects in one or more of the genes involved in these processes can directly result in DSBs [61]. Another potentially unique capability of CS may be that it can induce DSBs directly due to its large concentration of highly reactive organic and inorganic substances that either are free radicals or can generate free radicals within the cell that can subsequently attack DNA [62,63]. It has been speculated that multiple hydroxyl radicals produced in close proximity could directly cause DSBs and lead to ATM activation [50]. It is well documented that increased oxidative stress is a major mechanism by which CS causes airway damage that can lead to a host of pathogenic conditions including lung cancer [64,65], possible as a result of the induction of oxidatively derived DNA damage such as altered or mismatched bases, deletions and insertions, intra and inter-strand cross-links, or possibly DSBs. Additional research will be required to determine the primary mechanism(s) by which DSBs are formed in cells exposed to CS. Regardless of the mechanism by which CS induces DSBs, repair of this defect occurs through two major pathways, homologous recombination (HR) and non-homologous end-joining (NHEJ). In vertebrate cells, NHEJ is the dominant DSB repair mechanism, particularly in G_1_-phase cells [66]. However, reliance on NHEJ for accurate repair of CS-induced DSBs may be problematic for the damaged cell since this process is error-prone, often resulting in deletion of base pairs that can lead to the accumulation of defective DNA with each cell cycle following exposure to CS [67,68].

Assessing the sites of ATM activation and H2AX phosphorylation by UV fluorescence microscopy revealed different patterns of localization in CS-exposed cells. The sites of γH2AX localization had a distinct punctate pattern (Fig. 8C). This was evident despite the close proximity of individual foci with each other and the frequent overlap of their images. The presence of distinct foci of γH2AX IF is considered to be a reliable marker of DSBs [18-20]. In contrast to γH2AX, no distinct ATM-S1981^P ^IF foci were observed, and activated ATM was spread rather uniformly over the whole nucleoplasm (Fig. 7C). ATM activation in response to DSB formation, similar as H2AX phosphorylation, is purported to present as distinct ATM-S1981^P ^IF foci [3,17]. It is likely, therefore, that the observed pattern of ATM-S1981^P ^IF in A549 cells reflects, not only ATM activation in response to formation of DSBs, but also to other types of CS-induced DNA lesions that may have more global effects such as altering chromatin structure [3,17]. Such diverse spatial patterns of response in terms of nuclear ATM activation and H2AX phosphorylation are consistent with a variety of different DNA lesions generated by CS [39-48]. It has been proposed by Kitagawa et al. [17] that ATM phosphorylation may be triggered by "structural" changes in chromatin as a result of formation of DSBs or other types of DNA damage that leads to altered topological stress on the DNA double helix in the proximity of the damage. In support of this mechanism are the observations that chromatin condensation during mitosis [69,70] or premature chromosomes condensation [71] is accompanied by ATM activation. The altered topological stress on DNA in mitosis is reflected by DNA's increased propensity to undergo denaturation [72] and its sensitivity to single-strand specific endonucleases such as S1 or mung bean nuclease [73]. The observed CS-induced H2AX phosphorylation may also be mediated by the mechanism associated with NER and linked with the excision of DNA adducts generated by CS. This mechanism would be consistent with the recent findings of Marti et al [74] who reported H2AX phosphorylation in G_1 _cells upon exposure to UV-C light, which was abrogated in the NER mutant cells.

## Conclusion

ATM is a key element in the signal transduction pathways that detect DNA damage and orchestrate a multi-layered cellular response that includes recruiting DNA repair machinery, engaging cell cycle checkpoints, and increasing susceptibility to pro-apoptotic signals should DNA repair prove unsuccessful [2,4,6-9]. Its activation by CS provides evidence of extensive changes in the signal transmission pathways that are essential for cell survival and for protecting cells from neoplastic transformation. It would be expected, therefore, that genetic changes in terms of mutations in any of the individual components of ATM-associated pathways may constitute a significant risk factor in current or former smokers in terms of cancer development.

## Methods

### Cell culture and smoke treatment

A549 cells were purchased from American Type Culture Collection (ATCC no. CCL-185, Manassas, VA) and were cultured in Ham's F12K medium with 2 mM L-glutamine adjusted to contain 1.5 g/L sodium bicarbonate and supplemented with 10% fetal bovine serum (ATCC). All incubations were at 37°C in a humidified atmosphere of 5% CO_2 _in air. Cigarette smoke (CS) treatment was performed as follows: Dual-chambered slides (Nunc Lab-Tek II, VWR International, West Chester, PA) were seeded with 1 ml of 5 × 10^4 ^cells/ml cell suspension per chamber 48 hours before exposure and were typically at 70% confluency at the time of exposure to CS. The cell culture medium was replaced with 37°C Dulbecco's PBS (D-PBS) containing calcium and magnesium (BioSource, Rockville, MD) for the smoke exposure. Slide chamber covers were removed and the slides were placed in a smoke exposure chamber (20.6 cm × 6.7 cm × 6.3 cm -LxWxH). CS was generated under Federal Trade Commission (FTC)[75] smoking conditions (35 ± 0.3 cc puff, one puff every 60 seconds, 2-second puff duration with none of the ventilation holes blocked) using a KC 5 Port Smoker (KC Automation, Richmond, VA), from IM16 reference research cigarette (FTC values of 15.7 mg "tar" and 1.01 mg nicotine; Industry Monitor #16, Philip-Morris, Richmond VA,), 2R4F reference research cigarette (FTC values of 9.7 mg "tar" and 0.85 mg nicotine; University of Kentucky, Louisville, KY), or a leading commercially available U.S. cigarette brand with FTC values of 15 mg "tar" and 1.1 mg nicotine (Brand A). Cigarettes were smoked to within 3 mm of the filter tip. All cigarettes had been equilibrated at 23.9°C ± 1.1°C and 60% ± 2% relative humidity for a minimum of 24 hours and a maximum of 14 days. The smoke exposure chamber was designed to deliver smoke uniformly diluted with 5% CO_2 _in air and passed through the cell exposure chamber at a constant flow rate of 500 cc/min. Briefly, each 35 cc puff was first drawn into a 250 cc round chamber containing 5% CO_2 _in air and mixed via a stir bar. The standard smoke dilution used in most of our experiments was 35 cc delivered over 1 min in a 500 cc volume, and the intensity of exposure was varied by varying the length of time the cells spent in the exposure chamber. The time and distance that the smoke traveled from the end of the cigarette to the exposure chamber was minimized by using the shortest lengths of tubing possible between the parts of the apparatus.

Mock-exposed cells were treated under identical conditions as the exposed cells except for the absence of a cigarette in the smoking port. Following treatment or mock treatment, the D-PBS covering the cells was aspirated and replaced with 1 ml per chamber of fresh culture medium at 37°C. The cells were placed in the 37°C, 5% CO_2 _incubator and incubated for 1 hour. For immunocytochemical measurements the medium was aspirated and the cells fixed with 1% paraformaldehyde by gently rocking the slides at room temperature for 15 minutes. Following aspiration of the fixative, the chamber slides were disassembled and the slides submerged in 50 ml conical tubes filled with 70% ethanol for storage prior to analysis.

### Cell lysis

Following treatment, the culture medium was aspirated, cell monolayers were washed twice with cold D-PBS, 2 ml/dish, and 1 ml (per 10^6 ^cells) of RIPA cell lysis buffer (Pierce, Rockford, IL) containing protease and phosphatase inhibitors was added to the monolayer. Cells were scrapped into an eppendorf tube, vigorously pipetted, and left on ice for 25 minutes to allow complete lysis. The cell lysate was centrifuged at 10,000 × g for 25 minutes and the supernatant transferred to clean eppendorf tubes. All manipulations were done at 4°C. Protein concentration of the lysate was determined against a commercially available protein standard (Bio Rad, Hercules, CA) using a Bradford protein assay (Bio Rad detergent compatible assay reagent, Bio Rad).

### Western blot

The protein lysate was loaded on a 3–8% NuPAGE Tris-Acetate mini gel (Invitrogen, Carlsbad, CA) at a concentration of 10 μgs along with HiMark protein standard according to the manufacturing guidelines. The proteins were transferred to a PDVF membrane using a Bio Rad mini transfer apparatus (Bio Rad) overnight at 4°C using Bio Rad's transfer buffer containing 10% Methanol and 0.05% SDS. The membrane was blocked with T-20 protein free buffer (Pierce, Rockford, IL) for 1 hour at room temperature. The membrane was washed with TBST (Bio Rad) for 10 min (×3). The membrane was added to T-20 protein free buffer (Pierce) solution containing ATM-phospho, the primary antibody, (R&D systems, Minneapolis, MN) at a dilution of 1:300 and incubated at 4°C with gentle shaking, overnight. The membrane was washed with TBST for 10 min (×3). The membrane was then placed in T-20 protein free buffer solution containing Anti-Rabbit-HRP, secondary antibody, (Cell Signaling Technology, Beverly, MA) at a 1:2000 dilution and incubated for 1 hour at room temperature with gentle shaking. The membrane was washed with TBST for 10 min (×3). The blot was developed using Western lightning chemiluminescence (Perkins Elmore, Boston, MA).

### Caffeine and NU7026 treatment

Cells were pre-treated for one hour before exposure, concurrently treated during the 20 minute IM16 smoke exposure, and treated for one hour post-exposure with 4 mM caffeine (Sigma, St. Louis, MO) prepared as a 50 mM stock solution in PBS, or with 10 μM of the DNA-PKcs inhibitor NU7026 (Sigma) prepared as a 5 mM stock solution in DMSO, at which time cells were fixed onto dual-chamber slides with 1% paraformaldehyde. Final concentrations of caffeine and NU7026 was prepared in culture medium for the pre-treatment and post-exposure treatment in order to minimize shock to the cells while they were diluted to the appropriate concentration in D-PBS during the 20 min exposure to CS.

### Immunocytochemical detection of phosphorylated histone H2AX, activated ATM and caspase-3 activation

Cells were treated with CS and fixed as described above, then rinsed twice in PBS and incubated in 0.1% Triton X-100 (Sigma) in PBS for 15 min at room temperature, followed by incubation in a solution of 1% (w/v) bovine serum albumin (BSA; Sigma) in PBS for 30 min to suppress non specific antibody binding. The cells were then incubated in 100 μl volume of 1% BSA containing 1:200 dilution of phospho-specific (*Ser*-139) histone H2AX (γ-H2AX) mouse monoclonal antibody (mAb) (Millipore, Temecula, CA) or a 1:100 dilution of phospho-specific ATM (*Ser*-1981) mAb (Millipore). After overnight incubation at 4°C, the slides were washed twice with PBS and then incubated in 100 μl of 1:100 dilution of Alexa Fluor 488 goat anti-mouse IgG (H+L) (Invitrogen/Molecular Probes, Eugene, OR) for 40 min at room temperature in the dark. Parallel samples were incubated with 1:100 diluted anti-cleaved (activated) caspase-3 rabbit polyclonal antibody (Cell Signaling Technology) overnight at 4°C, washed twice with PBS and incubated with 1:100 diluted Alexa Fluor 488 goat anti-rabbit (IgG (H+L) (Invitrogen/Molecular Probes) for 40 min in room temperature in the dark. The cells were then counterstained with 1 μg/ml 4,6-diamidino-2-phenylindole (DAPI; Invitrogen/Molecular Probes) in PBS for 10 min. Each experiment was performed with an IgG control in which cells were labeled only with secondary antibody, Alexa Fluor 488 goat anti-mouse IgG (H+L) or Alexa Fluor 488 goat anti-rabbit IgG (H+L) without primary antibody incubation to estimate the extent of nonspecific binding of the secondary antibody to the cells.

### Measurement of cell fluorescence by Laser Scanning Cytometry

Cellular green phosphorylated histone H2AX, ATM or activated Caspase-3, and blue (DAPI) fluorescence emission was measured using a Laser Scanning Cytometer (LSC; iCys; CompuCyte, Cambridge, MA), utilizing standard filter settings; fluorescence was excited with 488-nm argon ion and violet diode lasers, respectively [76]. The intensities of maximal pixel and integrated fluorescence were measured and recorded for each cell. At least 3,000 cells were measured per sample.

### Statistical analysis

To compare the changes in γH2AX or ATM-S1981^P^immunofluorescence intensity (IF) the mean fluorescence intensity (integral values of individual cells) was calculated for cells in each phase of the cycle by gating G_1_, S and G_2_M cells based on differences in DNA content (DNA index, DI). The means of the fluorescence value for G_1_, S and G_2_M populations of cells in the IgG control groups were then subtracted from the respective means of the condensate or smoke-treated cells. As histone and DNA content double as cells proceed from G_1 _to G_2 _phase, the mean values of H2AX and ATM for the S and G_2_M cell populations were divided by 1.5 and 2.0, respectively, in order to express the degree of change in γH2AX or ATM-S1981^P ^IF, i.e., the increase in phosphorylated protein per unit of DNA. All experiments were run under identical instrument settings. Data is presented as mean γH2AX or ATM-S1981^P ^IF of each cell cycle compartment or where not indicated, of the entire population (G_1_, S and G_2_M). Each experiment was run in duplicate or triplicate. All experiments were repeated at least three times. Other details are presented in Figure legends.

## Abbreviations

ATM, *ataxia telangiectasia *mutated

ATR, ATM-and Rad-3 related kinase

CS, cigarette smoke

DNA-PKcs, DNA dependent protein kinase catalytic subunit

DSBs, DNA double-strand breaks

FTC, Federal Trade Commission

HR, homologous recombination

NAC, N-acetyl-L-cysteine

NER, nucleotide excision repair

NHBE, normal human bronchial epithelial cells

NHEJ, non-homologous end-joining

PIKKS, phosphoinositide 3-kinase related protein kinases

ROS, reactive oxygen species

## Authors' contributions

APA supervised the overall conduct of the research. APA, ZD, FT and EJ conceived of the study, participated in the study design, and provided continuous evaluation of all generated data. TT, XH, and DG carried out all of the experimental work in this study. FT performed the statistical analyses. APA, ZD, FT and EJ analyzed the data and wrote the manuscript. All authors have read and approved the final manuscript.

## References

[B1] Kastan MB, Lim DS (2000). The many substrates and functions of ATM. Nat Rev Mol Cell Biol.

[B2] Burma S, Chen BP, Murphy M, Kurimasa A, Chen DJ (2001). ATM phosphorylates histone H2AX in response to DNA double-strand breaks. J Biol Chem.

[B3] Bakkenist CJ, Kastan MB (2003). DNA damage activates ATM through intermolecular autophosphorylation and dimer dissociation. Nature.

[B4] Bakkenist CJ, Kastan MB (2004). Initiating cellular stress responses. Cell.

[B5] Helt CE, Cliby WA, Keng PC, Bambara RA, O'Reilly MA (2005). Ataxia telangiectasia mutated (ATM) and ATM and Rad3-related protein exhibit selective target specificities in response to different forms of DNA damage. J Biol Chem.

[B6] Lee JH, Paull TT (2005). ATM activation by DNA double-strand breaks through the Mre11-Rad50-Nbs1 complex. Science.

[B7] Paull TT, Lee JH (2005). The Mre11/Rad50/Nbs1 complex and its role as a DNA double-strand break sensor for ATM. Cell Cycle.

[B8] Abraham RT, Tibbetts RS (2005). Cell biology. Guiding ATM to broken DNA. Science.

[B9] Downs JA, Cote J (2005). Dynamics of chromatin during the repair of DNA double-strand breaks. Cell Cycle.

[B10] Sun Y, Jiang X, Chen S, Fernandes N, Price BD (2005). A role for the Tip60 histone acetyltransferase in the acetylation and activation of ATM. Proc Natl Acad Sci U S A.

[B11] Yong W, Bao S, Chen H, Li D, Sanchez ER, Shou W (2007). Mice lacking protein phosphatase 5 are defective in ATM-mediated cell cycle arrest. J Biol Chem.

[B12] Lavin MF, Kozlov S (2007). ATM activation and DNA damage response. Cell Cycle.

[B13] Bartkova J, Bakkenist CJ, Rajpert-De Meyts E, Skakkebaek NE, Sehested M, Lukas J, Kastan MB, Bartek J (2005). ATM activation in normal human tissues and testicular cancer. Cell Cycle.

[B14] Huang X, Halicka HD, Traganos F, Tanaka T, Kurose A, Darzynkiewicz Z (2005). Cytometric assessment of DNA damage in relation to cell cycle phase and apoptosis. Cell Prolif.

[B15] Kurose A, Tanaka T, Huang X, Halicka HD, Traganos F, Dai W, Darzynkiewicz Z (2005). Assessment of ATM phosphorylation on Ser-1981 induced by DNA topoisomerase I and II inhibitors in relation to Ser-139-histone H2AX phosphorylation, cell cycle phase, and apoptosis. Cytometry A.

[B16] Al Rashid ST, Dellaire G, Cuddihy A, Jalali F, Vaid M, Coackley C, Folkard M, Xu Y, Chen BP, Chen DJ, Lilge L, Prise KM, Bazett Jones DP, Bristow RG (2005). Evidence for the direct binding of phosphorylated p53 to sites of DNA breaks in vivo. Cancer Res.

[B17] Kitagawa R, Bakkenist CJ, McKinnon PJ, Kastan MB (2004). Phosphorylation of SMC1 is a critical downstream event in the ATM-NBS1-BRCA1 pathway. Genes Dev.

[B18] Rogakou EP, Pilch DR, Orr AH, Ivanova VS, Bonner WM (1998). DNA double-stranded breaks induce histone H2AX phosphorylation on serine 139. J Biol Chem.

[B19] Rogakou EP, Boon C, Redon C, Bonner WM (1999). Megabase chromatin domains involved in DNA double-strand breaks in vivo. J Cell Biol.

[B20] Sedelnikova OA, Rogakou EP, Panyutin IG, Bonner WM (2002). Quantitative detection of (125)IdU-induced DNA double-strand breaks with gamma-H2AX antibody. Radiat Res.

[B21] Banath JP, Olive PL (2003). Expression of phosphorylated histone H2AX as a surrogate of cell killing by drugs that create DNA double-strand breaks. Cancer Res.

[B22] Huang X, Okafuji M, Traganos F, Luther E, Holden E, Darzynkiewicz Z (2004). Assessment of histone H2AX phosphorylation induced by DNA topoisomerase I and II inhibitors topotecan and mitoxantrone and by the DNA cross-linking agent cisplatin. Cytometry A.

[B23] Olive PL (2004). Detection of DNA damage in individual cells by analysis of histone H2AX phosphorylation. Methods Cell Biol.

[B24] Albino AP, Huang X, Jorgensen E, Yang J, Gietl D, Traganos F, Darzynkiewicz Z (2004). Induction of H2AX phosphorylation in pulmonary cells by tobacco smoke: a new assay for carcinogens. Cell Cycle.

[B25] Halicka HD, Huang X, Traganos F, King MA, Dai W, Darzynkiewicz Z (2005). Histone H2AX phosphorylation after cell irradiation with UV-B: relationship to cell cycle phase and induction of apoptosis. Cell Cycle.

[B26] Park EJ, Chan DW, Park JH, Oettinger MA, Kwon J (2003). DNA-PK is activated by nucleosomes and phosphorylates H2AX within the nucleosomes in an acetylation-dependent manner. Nucleic Acids Res.

[B27] Cuadrado M, Martinez-Pastor B, Fernandez-Capetillo O (2006). "ATR activation in response to ionizing radiation: still ATM territory". Cell Div.

[B28] Ward IM, Minn K, Chen J (2004). UV-induced ataxia-telangiectasia-mutated and Rad3-related (ATR) activation requires replication stress. J Biol Chem.

[B29] Kurose A, Tanaka T, Huang X, Traganos F, Dai W, Darzynkiewicz Z (2006). Effects of hydroxyurea and aphidicolin on phosphorylation of ataxia telangiectasia mutated on Ser 1981 and histone H2AX on Ser 139 in relation to cell cycle phase and induction of apoptosis. Cytometry A.

[B30] Reitsema T, Klokov D, Banath JP, Olive PL (2005). DNA-PK is responsible for enhanced phosphorylation of histone H2AX under hypertonic conditions. DNA Repair (Amst).

[B31] Mukherjee B, Kessinger C, Kobayashi J, Chen BP, Chen DJ, Chatterjee A, Burma S (2006). DNA-PK phosphorylates histone H2AX during apoptotic DNA fragmentation in mammalian cells. DNA Repair (Amst).

[B32] Tanaka T, Kurose A, Huang X, Dai W, Darzynkiewicz Z (2006). ATM activation and histone H2AX phosphorylation as indicators of DNA damage by DNA topoisomerase I inhibitor topotecan and during apoptosis. Cell Prolif.

[B33] Stiff T, O'Driscoll M, Rief N, Iwabuchi K, Lobrich M, Jeggo PA (2004). ATM and DNA-PK function redundantly to phosphorylate H2AX after exposure to ionizing radiation. Cancer Res.

[B34] Wang H, Wang M, Wang H, Bocker W, Iliakis G (2005). Complex H2AX phosphorylation patterns by multiple kinases including ATM and DNA-PK in human cells exposed to ionizing radiation and treated with kinase inhibitors. J Cell Physiol.

[B35] Albino AP, Huang X, Jorgensen ED, Gietl D, Traganos F, Darzynkiewicz Z (2006). Induction of DNA double-strand breaks in A549 and normal human pulmonary epithelial cells by cigarette smoke is mediated by free radicals. Int J Oncol.

[B36] Blasina A, Price BD, Turenne GA, McGowan CH (1999). Caffeine inhibits the checkpoint kinase ATM. Curr Biol.

[B37] Hollick JJ, Golding BT, Hardcastle IR, Martin N, Richardson C, Rigoreau LJ, Smith GC, Griffin RJ (2003). 2,6-disubstituted pyran-4-one and thiopyran-4-one inhibitors of DNA-Dependent protein kinase (DNA-PK). Bioorg Med Chem Lett.

[B38] Oricchio E, Saladino C, Iacovelli S, Soddu S, Cundari E (2006). ATM is activated by default in mitosis, localizes at centrosomes and monitors mitotic spindle integrity. Cell Cycle.

[B39] Nakayama T, Kaneko M, Kodama M, Nagata C (1985). Cigarette smoke induces DNA single-strand breaks in human cells. Nature.

[B40] Einhaus M, Holz O, Meissner R, Krause T, Warncke K, Held I, Scherer G, Tricker AR, Adlkofer F, Rudiger HW (1994). Determination of DNA single-strand breaks in lymphocytes of smokers and nonsmokers exposed to environmental tobacco smoke using the nick translation assay. Clin Investig.

[B41] Leanderson P, Tagesson C (1994). Cigarette tar promotes neutrophil-induced DNA damage in cultured lung cells. Environ Res.

[B42] Villard PH, Seree EM, Re JL, De Meo M, Barra Y, Attolini L, Dumenil G, Catalin J, Durand A, Lacarelle B (1998). Effects of tobacco smoke on the gene expression of the Cyp1a, Cyp2b, Cyp2e, and Cyp3a subfamilies in mouse liver and lung: relation to single strand breaks of DNA. Toxicol Appl Pharmacol.

[B43] Hecht SS (1999). DNA adduct formation from tobacco-specific N-nitrosamines. Mutat Res.

[B44] Tsuda S, Matsusaka N, Ueno S, Susa N, Sasaki YF (2000). The influence of antioxidants on cigarette smoke-induced DNA single-strand breaks in mouse organs: a preliminary study with the alkaline single cell gel electrophoresis assay. Toxicol Sci.

[B45] Ziegel R, Shallop A, Jones R, Tretyakova N (2003). K-ras gene sequence effects on the formation of 4-(methylnitrosamino)-1-(3-pyridyl)-1-butanone (NNK)-DNA adducts. Chem Res Toxicol.

[B46] Paz-Elizur T, Krupsky M, Blumenstein S, Elinger D, Schechtman E, Livneh Z (2003). DNA repair activity for oxidative damage and risk of lung cancer. J Natl Cancer Inst.

[B47] DeMarini DM (2004). Genotoxicity of tobacco smoke and tobacco smoke condensate: a review. Mutat Res.

[B48] Wu HJ, Chi CW, Liu TY (2005). Effects of pH on nicotine-induced DNA damage and oxidative stress. J Toxicol Environ Health A.

[B49] Mills KD, Ferguson DO, Alt FW (2003). The role of DNA breaks in genomic instability and tumorigenesis. Immunol Rev.

[B50] Ismail IH, Nystrom S, Nygren J, Hammarsten O (2005). Activation of ataxia telangiectasia mutated by DNA strand break-inducing agents correlates closely with the number of DNA double strand breaks. J Biol Chem.

[B51] Vilenchik MM, Knudson AG (2003). Endogenous DNA double-strand breaks: production, fidelity of repair, and induction of cancer. Proc Natl Acad Sci U S A.

[B52] Beckman KB, Ames BN (1997). Oxidative decay of DNA. J Biol Chem.

[B53] Gorbunova V, Seluanov A (2005). Making ends meet in old age: DSB repair and aging. Mech Ageing Dev.

[B54] Husgafvel-Pursiainen K (2004). Genotoxicity of environmental tobacco smoke: a review. Mutat Res.

[B55] Huang X, Tanaka T, Kurose A, Traganos F, Darzynkiewicz Z (2006). Constitutive histone H2AX phosphorylation on Ser-139 in cells untreated by genotoxic agents is cell-cycle phase specific and attenuated by scavenging reactive oxygen species. Int J Oncol.

[B56] Wakeman TP, Kim WJ, Callens S, Chiu A, Brown KD, Xu B (2004). The ATM-SMC1 pathway is essential for activation of the chromium[VI]-induced S-phase checkpoint. Mutat Res.

[B57] Kitagawa R, Kastan MB (2005). The ATM-dependent DNA damage signaling pathway. Cold Spring Harb Symp Quant Biol.

[B58] D'Agostini F, Balansky RM, Izzotti A, Lubet RA, Kelloff GJ, De Flora S (2001). Modulation of apoptosis by cigarette smoke and cancer chemopreventive agents in the respiratory tract of rats. Carcinogenesis.

[B59] Perlow RA, Kolbanovskii A, Hingerty BE, Geacintov NE, Broyde S, Scicchitano DA (2002). DNA adducts from a tumorigenic metabolite of benzo[a]pyrene block human RNA polymerase II elongation in a sequence- and stereochemistry-dependent manner. J Mol Biol.

[B60] Quievryn G, Peterson E, Messer J, Zhitkovich A (2003). Genotoxicity and mutagenicity of chromium(VI)/ascorbate-generated DNA adducts in human and bacterial cells. Biochemistry.

[B61] Roos WP, Kaina B (2006). DNA damage-induced cell death by apoptosis. Trends Mol Med.

[B62] Green CR, Rodgman A (1996). The tobacco chemists' research conferences: a half century of advances in analytical methodology of tobacco and its products.. Recent Advances in Tobacco Science.

[B63] IARC (International Agency for Research on Cancer) (2003). Tobacco Smoking and Involuntary Smoking: Monograph on the Evaluation of Carcinogenic Risk of Chemicals to Humans.

[B64] Ballaz S, Mulshine JL (2003). The potential contributions of chronic inflammation to lung carcinogenesis. Clin Lung Cancer.

[B65] van der Vaart H, Postma DS, Timens W, ten Hacken NH (2004). Acute effects of cigarette smoke on inflammation and oxidative stress: a review. Thorax.

[B66] Watrin E, Peters JM (2006). Cohesin and DNA damage repair. Exp Cell Res.

[B67] Pastwa E, Blasiak J (2003). Non-homologous DNA end joining. Acta Biochim Pol.

[B68] Jeggo PA, Lobrich M (2005). Artemis links ATM to double strand break rejoining. Cell Cycle.

[B69] Ichijima Y, Sakasai R, Okita N, Asahina K, Mizutani S, Teraoka H (2005). Phosphorylation of histone H2AX at M phase in human cells without DNA damage response. Biochem Biophys Res Commun.

[B70] Tanaka T, Halicka HD, Huang X, Traganos F, Darzynkiewicz Z (2006). Constitutive histone H2AX phosphorylation and ATM activation, the reporters of DNA damage by endogenous oxidants. Cell Cycle.

[B71] Huang X, Kurose A, Tanaka T, Traganos F, Dai W, Darzynkiewicz Z (2006). Sequential phosphorylation of Ser-10 on histone H3 and ser-139 on histone H2AX and ATM activation during premature chromosome condensation: relationship to cell-cycle phase and apoptosis. Cytometry A.

[B72] Juan G, Pan W, Darzynkiewicz Z (1996). DNA segments sensitive to single-strand-specific nucleases are present in chromatin of mitotic cells. Exp Cell Res.

[B73] Darzynkiewicz Z, Traganos F, Sharpless T, Melamed MR (1977). Different sensitivity of DNA in situ in interphase and metaphase chromatin to heat denaturation. J Cell Biol.

[B74] Marti TM, Hefner E, Feeney L, Natale V, Cleaver JE (2006). H2AX phosphorylation within the G1 phase after UV irradiation depends on nucleotide excision repair and not DNA double-strand breaks. Proc Natl Acad Sci U S A.

[B75] Register F (1967).

[B76] Bedner E, Burfeind P, Gorczyca W, Melamed MR, Darzynkiewicz Z (1997). Laser scanning cytometry distinguishes lymphocytes, monocytes, and granulocytes by differences in their chromatin structure. Cytometry.

[B77] Tanaka T, Kajstura M, Halicka HD, Traganos F, Darzynkiewicz Z (2007). Constitutive histone H2AX phosphorylation and ATM activation are strongly amplified during mitogenic stimulation of lymphocytes. Cell Prolif.

